# Lipase production from *Bacillus safensis* VC-6 isolated from the volcanic region of Copahue: optimization and functional genomic insights

**DOI:** 10.3389/fmicb.2025.1621262

**Published:** 2025-08-20

**Authors:** Valeria Foronda, Valeria Castellanos, Claudia Hoepfner, Daniel Guzmán, Héctor Guzmán, Jerry L. Solis

**Affiliations:** ^1^Centro de Biotecnología, Facultad de Ciencias y Tecnología, Universidad Mayor de San Simón, Cochabamba, Bolivia; ^2^GEMA Center for Genomics, Ecology and Environment, Faculty of Interdisciplinary Studies, Universidad Mayor, Santiago, Chile

**Keywords:** lipase production, *Bacillus safensis*, enzyme, biotechnology, functional genomic, bioreactor, extremophiles

## Abstract

Extremophilic microorganisms produce highly stable and industrial-grade enzymes with enhanced performance. Thermostable enzymes, such as lipases that catalyze the hydrolysis and esterification of lipids, are of great industrial interest due to their stability and efficacy under harsh conditions, making them ideal for applications in biotechnology, pharmaceuticals, and cosmetics. Lipase production from various microorganisms is well-studied. However, optimization studies remain limited for lipases sourced from halotolerant bacteria, such as *Bacillus safensis* strain VC-6, known to grow above 10% (w/v) NaCl and 50°C. The limited research on optimizing these enzymes prevents their widespread adoption in industries requiring high thermostability and solvent tolerance. This study optimized the production of thermostable and halotolerant lipases using the extremophilic strain VC-6, isolated from samples from the Copahue Volcano, Chile. Strain VC-6 was selected from five candidate strains due to its stable growth within simple culture media and positive results in qualitative lipase activity assays. In the initial phases, VC-6 demonstrated superior potential for lipase production. Growth conditions were optimized using a heterotrophic medium supplemented with 2% (w/v) NaCl, 2% (v/v) glycerol, and pH 6 at 37°C. Lipase production was maximized based in the previous medium supplemented with 1% (w/v) yeast extract, 0.5% (w/v) KCl, 3% (v/v) sunflower oil, 2% (v/v) glycerol, and pH 8 at 37°C. Extracellular lipase activity was assessed, and enzyme recovery was facilitated through precipitation methods. Lipase activity was quantified in a batch bioreactor under controlled conditions achieving a maximum enzymatic activity of 12.83 U mL^−1^ at 16 h of cultivation, correlated with the exponential growth phase of the bacteria. Genetic identification (16S rRNA gene) confirmed that strain VC-6 belongs to the *Bacillus* genus, sharing 99.93% similarity with *Bacillus safensis*. Genomic analysis revealed the presence of key genes related to lipase production, including YtpA (phospholipase), LipC (germination lipase), and a thermostable monoacylglycerol lipase. These genes likely explain the observed peaks of enzymatic activity during the fermentation process, with distinct activity observed at different time points. This study highlights the potential of *Bacillus safensis* strain VC-6 as a promising source of thermostable and halotolerant lipases. The integration of optimized bioprocess conditions and genomic-based understanding establishes a solid groundwork for the future industrial exploitation of these biocatalysts under extreme environmental conditions. The optimization of growth conditions and the identification of critical genes related to lipase production further enhance the potential for scaling up production processes in biotechnological applications.

## 1 Introduction

Enzymes are specialized proteins involved in the catalysis of reactions within living systems, playing a fundamental role in numerous biochemical processes. Among them, lipases constitute a class of hydrolytic enzymes capable of catalyzing the conversion of triacylglycerols into free fatty acids and glycerol, facilitating this reaction at the lipid–water interface due to the low solubility of their substrates. This catalytic capacity grants lipases outstanding biotechnological value, making them versatile and specific biocatalysts widely employed in food, pharmaceutical, chemical, and biofuel industries, as well as in bioremediation processes targeting environments contaminated with lipid compounds (Han and Sari, 2024; Fatima et al., 2021).

Thermostable and halotolerant lipases hold strategic value in industrial applications that require extreme conditions, such as elevated temperatures or high salinity. These properties are frequently associated with extremophilic microorganisms, whose adaptability to hostile environments often correlates with the catalytic stability of their enzymes (Espina et al., 2022). In this regard, extreme environments such as hydrothermal zones, deserts, salt flats, glaciers, deep oceans, and volcanic regions represent ecosystems of high biotechnological interest due to their unique environmental conditions and microbial diversity (Cowan et al., 2015).

The volcanic region of Copahue-Chile, located in the Andes Mountains, stands out for its unique geothermal conditions, high UV radiation, considerable thermal variability, and elevated altitude. These characteristics make it a natural reservoir of extremophilic microorganisms with enzymatic potential. The exploration of this microbial biodiversity enables the identification of strains capable of producing stable and efficient enzymes, with relevant applications in sustainable industrial processes (Urbieta et al., 2015).

Despite the recognized potential of such environments, comprehensive studies optimizing lipase production and linking it to genomic features in strains like those from Copahue are limited. Having isolated several bacterial strains with potential applications, this study focuses on VC-6 strain, an extremophilic microorganism isolated from the volcanic environment of Copahue. The strain VC-6 exhibited high lipolytic potential and efficient growth in lipase induction media. Optimization of lipase production under controlled conditions was addressed, specifically in a batch reactor system. Furthermore, the genomic characterization of the strain was conducted to identify key genes associated with lipase synthesis, aiming to establish culture parameters that maximize enzyme yield. The optimization of these processes represents a significant step toward the development of sustainable biocatalysts capable of replacing conventional chemical catalysts, thereby contributing to a more efficient and environmentally friendly industry.

The novelty of this study lies in the integration of medium optimization with functional genomic analysis in an extremophilic *Bacillus safensis* strain isolated from a volcanic environment. To the best of our knowledge, this is one of the first comprehensive characterizations of *B. safensis* lipases that simultaneously reports the presence of genes such as *YtpA, LipC*, and a putative thermostable MAG-type lipase, while correlating these genomic features with enzymatic activity peaks observed under bioreactor conditions. This integrated approach strengthens the biotechnological relevance of the strain and sets a foundation for further exploitation of its lipolytic system.

## 2 Materials and methods

### 2.1 Strain isolation and selection

The microbial samples used in this study were originally collected during a microbial diversity survey in the volcanic region of Copahue, Chile in 2018. The samples were kindly provided by Prof. Jaime Melendez (former Associate Professor at Pontificia Universidad Católica de Chile). The basal medium for isolation, maintenance, and seed of 12 microbial strains contained (% w/v): yeast extract, 1; peptone, 0.5; glucose, 0.1; MgSO_4_·H_2_O, 0.025; KCl, 0.050; CaCl_2_, 0.009; NaBr, 0.006; and agar, 2. Medium pH was adjusted to 7 using 1 mol L^−1^ NaOH. The strains were cultured using the streaked plate method, ensuring adequate colony growth.

Once the microbial collection was isolated, lipase production potential was screened using a modified lipase-inducing medium in plates. The modified medium contained the same composition as the basal medium but supplemented with 1% (v/v) olive oil and 0.001% (p/v) rhodamine β, while glucose was removed from the medium. The strain colony that showed the highest fluorescent intensity under UV light was selected for further lipase-related analyses.

### 2.2 Bacterial morphological characterization

The selected strain was morphologically characterized at both micro- and macro-scales. Gram staining was performed following the standardized protocol (Smibert and Krieg, 1994). After staining, colony morphology was assessed in accordance with the guidelines outlined in the ASM protocol (Carroll et al., 2019).

### 2.3 Optimization of growth culture conditions

The selected strain was cultured in modified basal media to determine optimal growth conditions. Initially, the selected strain was grown in 250 mL shake flasks with 60 mL seed medium at 37°C and 200 rpm in a rotary shaker incubator (New Brunswick Scientific, NJ, USA). Optical density at 600 nm (OD_600_) was measured every 2 h to monitor cell growth and identify the time of maximum biomass accumulation. Subsequently, a propagation step was carried out with 2 mL of the previous culture, which was inoculated in 500 mL shake flasks with 125 mL of seed medium. Temperature, pH, type of triglyceride-based carbon source (TG), and its concentration were defined as the independent variables (IV), while the basal medium composition was used as the starting point for the study. The enlisted independent variables were sequentially and systematically tested as described in Table 1. A completely randomized design (CRD) approach was applied within each IV, having 3 repetitions per level.

**Table 1:** Experimental parameters following a sequential optimum approach.

**Independent variable (IV)**	**Units**	**Levels**
Temperature	°C	4, 24, 37, 50, 80
pH	–	5.0, 6.0, 7.0, 8.0, 9.0
Triglyceride source	% (w/v)	Soybean oil (*Glycine max*)
		Sunflower oil (*Helianthus annuus*)
		Soybean and sunflower oil (1:1)
		Olive oil
		Cusi oil (*Attalea speciosa*)
		Castor oil (*Ricinus communis*)
		Used canola oil (*Brassica napus*)
		Mayonnaise
Triglyceride source concentration (selected)	% (w/v)	0.5, 1.0, 1.5, 2.0, 2.5

The experimental framework is summarized in Table 1, which outlines the units and levels tested for each IV. Temperatures ranged from 4 to 80°C, and pH levels from 5.0 to 9.0. A variety of carbon sources rich in triglycerides were evaluated at 1% (w/v), including soybean oil, sunflower oil, a mixture of 3% (w/v) soybean and 2% (w/v) sunflower oil, olive oil, cusi oil, castor oil, used canola oil, and mayonnaise. Once the most suitable lipid source was identified, its concentration was further optimized by testing levels between 0.5% and 2.5% (w/v).

The dependent variable (DV) was measured by correlation of cell growth and broth optical density (OD_600_) at 600 nm (Myers et al., 2013). In parallel, cell dry weight was determined for each test (Godbey, 2021). A sample of 1.5 mL was pipetted from the culturing flask into a microtube. The sample was centrifuged up to 10,000 × g for 10 min at room temperature (RT) for biomass precipitation. The formed pellet at the bottom of the microtube was washed with 0.5 mL of distilled water, centrifuged again up to 10,000 × g for 10 min at RT, and dried at 80°C without the supernatant until constant weight was recorded.

Once the optimum growth conditions were determined, the following experiments were focused on the lipid-based carbon source and the construction of a growth curve. The lipid-based carbon source concentration for optimal cell growth was assayed at concentrations of 0.5%, 1.0%, 1.5%, 2.0%, and 2.5% (w/w). The optimized culture medium and growth conditions were applied to assemble the growth curve of the selected strain. The strain was cultivated in a 100 mL shake flask for 24 h and subsequently inoculated into a 500 mL shake flask. The volume ratio of culture medium to flask was kept at 1:4 to ensure thorough mixing. During 32 h incubation at 37°C, samples were taken every 2 h to monitor cell growth by OD_600_ and cell dry weight. The samples were later centrifuged at 10,000 × g for 10 min, and the supernatant was used to quantify total protein content using the Bradford method (Bradford, 1976; Kruger, 1994).

### 2.4 Lipase-inducing media selection

Optimum bacterial growth conditions (previously described) were established as the starting conditions for lipase production tests. Initially, the location of the major lipase concentration was evaluated. Cultures were separated by centrifuge at 10,000 × g for 10 min into supernatant, precipitated cellular pellet, and whole-culture fractions (Bharathi and Rajalakshmi, 2019; Mahnashi et al., 2022). Following, the supernatant (extracellular fraction) was recovered, and the cell pellet (intracellular fraction) was washed and resuspended in the original culture volume. The obtained fractions were analyzed using the p-NPP assay to quantify lipase activity.

Lipase activity was assessed using p-nitrophenyl palmitate (p-NPP) as substrate, in accordance with the protocol described by Guo et al. (2015). First, a volume of 1,280 μL of 0.05 mol L-1 phosphate buffer (pH 8.0) was pipetted into a 2 mL microtube. Subsequently, 160 μL of 0.03 mol L^−1^ p-NPP solution (prepared in 99% (v/v) ethanol solution) was added. After 5 min at RT, 160 μL of centrifuged sample (supernatant with enzymatic activity) was added to initiate the hydrolysis reaction, followed by gentle mixing. The reaction was allowed to proceed at RT for 20 min. Enzymatic activity was stopped by the addition of 400 μL of ice-cold 1 mol L^−1^ Na_2_CO_3_, which induces enzyme denaturation. Upon microtube cooling, the reaction mixture was centrifuged at 13,000 × g for 10 min. The resulting supernatant was transferred to a quartz cuvette, and the absorbance was measured at 318 nm using a UV-Visible spectrophotometer. The concentration of released p-nitrophenol (pNP) was quantified based on a previously established calibration curve. Lipase activity was expressed in international units (U), where one unit corresponds to the amount of enzyme required to release 1 μmol of pNP per minute per mL of extract under the assay conditions.

A sequential experimental approach was used to evaluate the effects of various independent variables on lipase biosynthesis, including type and combination of lipid-based carbon sources, nitrogen sources, and inorganic salt. Complimenting, initial culture medium pH was also added to the tests. All experiments were conducted in triplicate under a completely randomized design (CRD). Experimental treatments and levels are shown in Table 2. Enzymatic activity and protein concentration were determined as described above.

**Table 2:** Experimental design for lipase production optimization.

**Independent variable (IV)**	**Units**	**Levels**
Triglyceride-based carbon source	2% (v/v)	Sunflower oil (*Helianthus annuus*)
		Soybean oil (*Glycine max*)
		Mixed soybean and sunflower oils (1:1)
		Olive oil (*Olea europaea*)
		Cusi oil (*Attalea speciosa*)
		Castor oil (*Ricinus communis*)
		Used canola oil (*Brassica napus*)
		Used sunflower oil
		Glycerol
		Mayonnaise
		Fresh milk
		Curdled milk
Carbon source combinations	% (v/v)	3% glycerol + 2% sunflower oil
		3% sunflower oil + 2% glycerol
		2% sunflower oil
		3% glycerol + 2% olive oil
		3% olive oil + 2% glycerol
		2% olive oil
		3% glycerol
Nitrogen source	1% (w/v)	Yeast extract
		Peptone
		Ammonium chloride
		Potassium nitrate
		Magnesium nitrate
Inorganic salt	2% (w/v)	Sodium phosphate
		Monopotassium phosphate
		Magnesium sulfate heptahydrate (MgSO4·7H_2_O)
		Zinc sulfate heptahydrate (ZnSO4·7H_2_O)
		Ammonium sulfate [(NH4)_2_SO4]
		Barium chloride dihydrate (BaCl_2_·2H_2_O)
		Calcium chloride (CaCl_2_)
		Magnesium chloride hexahydrate (MgCl_2_·6H_2_O)
		Potassium chloride (KCl)
		Lithium chloride (LiCl)
		Sodium chloride (NaCl)
Salt concentration (highest response)	% (w/v)	0.5, 1.0, 1.5, 2.0, 2.5
pH	–	4.0, 5.0, 6.0, 7.0, 8.0

Normality and homogeneity of variances were assessed using Shapiro-Wilk and Levene's tests, respectively. When these assumptions were met, analysis of variance (ANOVA) was applied. Otherwise, non-parametric comparisons were conducted using Kruskal–Wallis and the Mann–Whitney *U*-tests. Statistical analyses were performed in R version 4.3.2 (R Core Team, 2013). Differences were considered statistically significant at *p* < 0.05.

Different lipase-inducing culture media were tested, each supplemented with individual lipidic carbon sources such as olive oil, glycerol, and other triglyceride-rich substrates (Bharathi and Rajalakshmi, 2019). These carbon sources were evaluated at 2% (w/v), and lipase activity was determined in the crude enzymatic extract. Growth was monitored by OD_600_ and cell dry weight. The medium yielding the highest enzymatic activity was selected for subsequent optimization steps.

Post-screening, selected carbon sources were tested in binary combinations to assess potential synergistic effects—particularly between glycerol, sunflower oil, and olive oil (Subroto et al., 2020). Cultures were maintained under standardized conditions, and treatments were compared based on enzyme activity and biomass yield.

Further optimization of lipase production involved assessing the effects of different organic (yeast extract, peptone) and inorganic (ammonium chloride, potassium nitrate, magnesium nitrate) nitrogen sources, each supplied at 1% (w/v) (Chandra et al., 2020; de Almeida et al., 2013; Gupta et al., 2005). In parallel, the effect of mono- and divalent cations was assessed by supplementing inorganic salts at 2% (w/v), including phosphates, sulfates, and chlorides containing a wide range of mono- and divalent cations (e.g., Na^+^, K^+^, Mg^2^^+^, Ca^2^^+^, Zn^2^^+^, Li^+^, Ba^2^^+^). The salt that resulted in the greatest increase in lipase activity was selected for further evaluation by varying its concentration from 0.5 to 2.5% (w/v) under the same culture conditions (Kaar et al., 2003; Bora and Bora, 2012).

The effect of the initial medium pH on lipase biosynthesis was also investigated by adjusting the culture to pH values from 4.0 to 8.0 with 1 mol L^−1^ HCl and 1 mol L^−1^ NaOH. After 24 h incubation, lipase activity and growth were measured to determine the optimal pH for enzyme production (Bora and Bora, 2012; Bakir and Metin, 2017).

### 2.5 Genetic identification and characterization

#### 2.5.1 Genomic DNA isolation and purification

For the identification of the isolated microorganism, genomic DNA was extracted using the PureLink^®^ Genomic DNA Mini Kit (Invitrogen), following the manufacturer's instructions. The quality and integrity of the extracted DNA were verified via 1% (w/v) agarose gel electrophoresis, and its concentration was quantified using a Qubit™ 4.0 fluorometer (ThermoFisher Scientific).

Subsequently, the 16S rRNA gene was amplified through polymerase chain reaction (PCR), employing the universal primers 27F (5′-AGAGTTTGATCCTGGCTCAG-3′) and 1492R (5′-TACGGYTACCTTGTTACGACTT-3′). The PCR conditions included an initial denaturation at 95°C for 30 s, annealing at 53°C for 30 s, and extension at 72°C for 1 min. The resulting PCR products were purified using the PureLink™ Quick PCR Purification Kit (Invitrogen), according to the manufacturer's protocol.

Purified products were again analyzed through 1% (w/v) agarose gel electrophoresis to confirm DNA integrity. DNA concentration was measured with the Qubit™ 4.0 fluorometer, ensuring a final concentration above 20 ng, as required for sequencing. A final purification step was conducted using the BigDye™ X-Terminator Purification Kit prior to sequencing reactions.

#### 2.5.2 Molecular identification

For sequencing, primers targeting the full length of the 16S rRNA gene, covering the V1V9 regions, were used on an Applied Biosystems 310 genetic analyzer. The primers included: 27F (5′-AGAGTTTGATCMTGGCTCAG-3′), 1492R (5′-TACGGYTACCTTGTTACGACTT-3′), and 515F (5′-GTGCCAGCMGCCGCGGTAA-3′).

Initial sequence analyses were performed using the EzBioCloud online platform (https://www.ezbiocloud.net/), a specialized database for prokaryotic identification via 16S rRNA gene sequences. The resulting fragments were assembled using the MEGA X software with Muscle as the alignment tool.

For further phylogenetic resolution, a full-length 16S sequence was compared using the BLASTn algorithm against the SILVA database through local alignment. Multiple sequence alignment was conducted using MAFFT, retaining only sequences showing ≥97% similarity. A phylogenetic tree was constructed using the IQ-TREE software, based on the maximum likelihood (ML) method, and visualized with FigTree.

#### 2.5.3 Whole-genome sequencing, assembly, and functional annotation

Whole-genome sequencing was performed using the Oxford Nanopore platform. Raw read quality was initially assessed using NanoPlot to evaluate read length and quality distributions. Reads were subsequently filtered and trimmed using NanoFilt, applying a minimum average quality score of 8, a minimum read length of 1,000 bp, and cropping the first 75 bases to remove potential low-quality regions. Quality-filtered reads were re-evaluated. Genome assembly was performed using Unicycler v0.4.7 with default parameters. The finalized genome assembly was then functionally annotated using Prokka, specifying the genus Bacillus, and the species safensis to ensure taxonomically informed gene prediction and functional assignments.

### 2.6 Lipase production under controlled bioreactor conditions

Lipase production by *Bacillus safensis* VC-6 strain was carried out in a 3-liter stirred-tank bioreactor (Winpact Evo Fermentation System, FS-01 series), operated under previously optimized controlled conditions, with the aim of maximizing enzymatic activity.

The determined optimum culture medium was prepared in a total volume of 2.5 L using the optimized lipase production medium and was sterilized together with the bioreactor prior to the initiation of the process. The system was equipped with a Rushton-type for thorough mixing and efficient oxygen transfer. The bioreactor was inoculated with 250 mL of 12 h culture, timing when the cells were in the exponential growth phase for inoculation.

Throughout the fermentation process, the bioreactor's automated control system maintained a constant temperature of 37°C and a pH of 8.0. Automated addition of 5 mol L^−1^ NaOH and 5 mol L^−1^ HCl via peristaltic pumps allowed pH control. Agitation was set at 400 rpm, and aeration was sustained through the continuous injection of sterile air at a flow rate of 1 L min^−1^, with the aim of maintaining dissolved oxygen at 60% saturation throughout the entire fermentation.

The bioprocess lasted 24 h, during which 3-milliliter samples were collected hourly through a sterile sampling line integrated into the system. Each sample was analyzed to monitor bacterial growth, total protein concentration, and enzymatic activity.

### 2.7 Lipase activity control in optimized production medium

Internal controls were established to monitor the lipase activity produced by strain VC-6 in an optimized production medium. A commercial lipase (Sigma-Aldrich 62301) served as the positive control, and sterile production medium was used as the negative control. Total protein concentration and enzymatic activity, measured through the hydrolysis of p-NPP, were analyzed over a 0–28 h incubation period.

## 3 Results

### 3.1 Isolation and selection of lipase-producing microorganisms

Strain VC-6 outperformed the collection of strains by the most intense fluorescence under UV light and was selected for the following studies. The bacterial strain VC-6 exhibited favorable physiological traits for biotechnological applications, including rapid growth (18 h to reach exponential growth phase), tolerance to 2% (w/v) NaCl, and consistent lipase activity in the presence of 0.5% (v/v) olive oil under mild conditions (pH 7 and 37°C).

### 3.2 Morphological characterization of the strain

**Figure 1:**
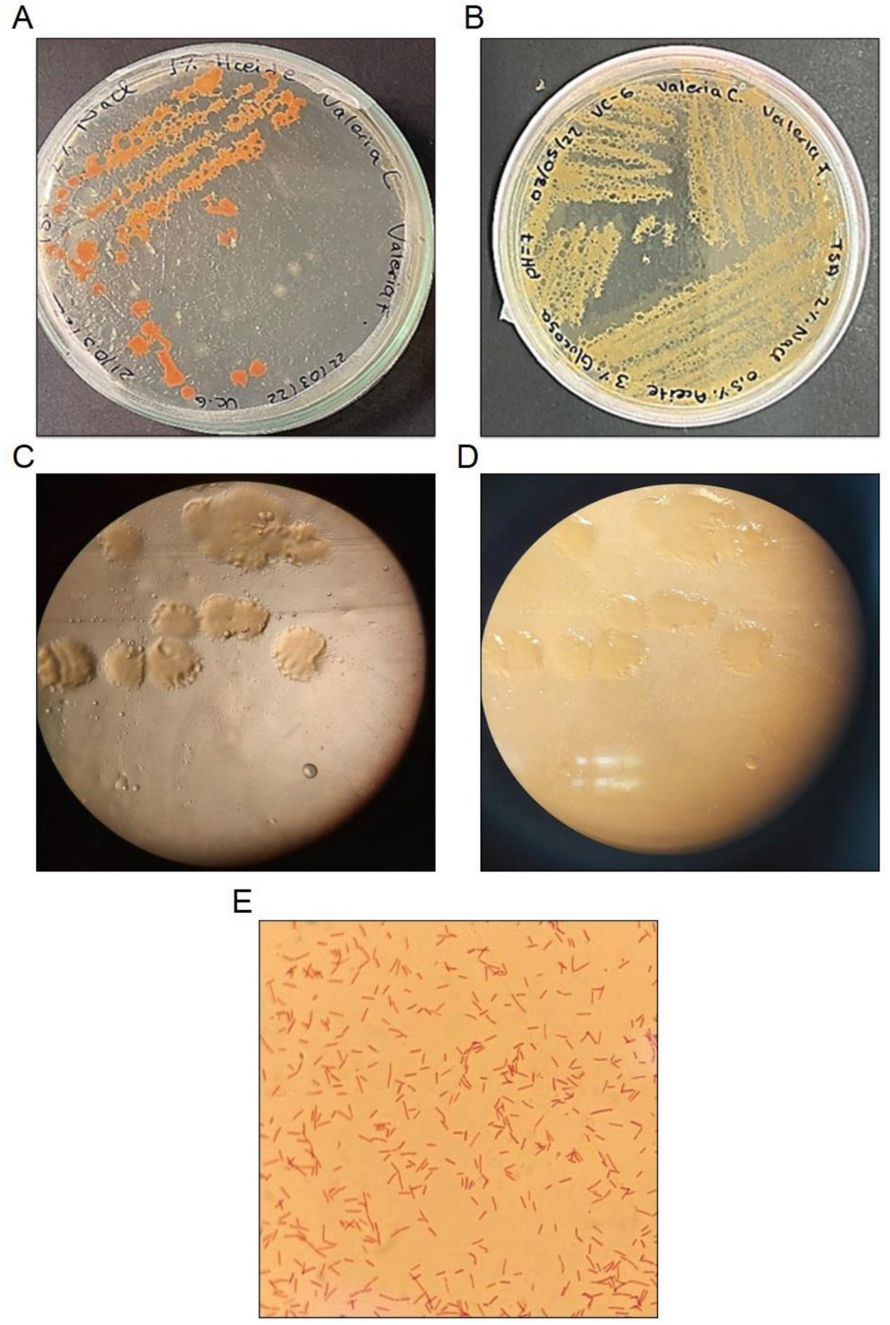
Morphological characterization of *Bacillus safensis* VC-6. *Bacillus safensis* VC-6 **(A)** growth at room temperature, **(B)** growth at 37°C, **(C, D)** colony morphology observed under a stereomicroscope, and **(E)** Gram staining.

Colonies of strain VC-6 appeared irregular in shape, with undulate margins and an elevated surface. Under transmitted light, they were translucent and exhibited a bright reflective appearance (Figures 1C, D). Colony pigmentation was orange at RT (Figure 1A) and cream-colored at 37°C (Figure 1B), as observed in Figures 1A, B, respectively. Microscopic observation revealed rod-shaped Gram-positive cells (Figure 1E).

### 3.3 Optimization of culture conditions

#### 3.3.1 Carbon source variation

**Figure 2:**
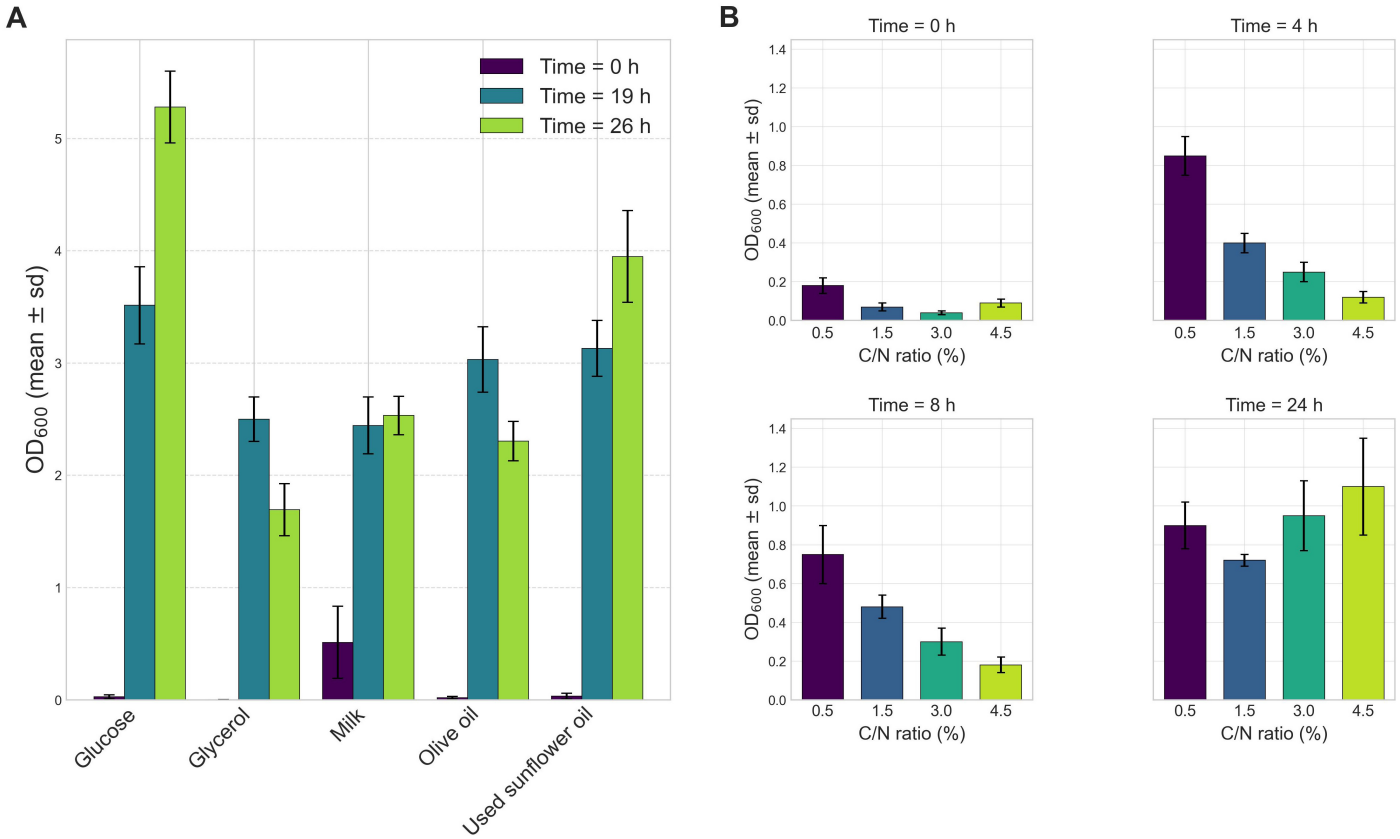
Effect of carbon sources and concentration on the growth of strain VC-6. **(A)** Glucose provided the highest growth reaching 4.91 OD_600_ after 26.3 h. **(B)** Culture corresponding to 4.5% C/N ratio showed the highest OD_600_ of 1.13 after 24 h.

Quantitative analysis of bacterial growth was performed using different carbon sources at a concentration of 1% (w/v): milk, glucose, olive oil, waste oil, and glycerol. Strain VC-6 exhibited the highest growth when glucose was used as the sole carbon source, reaching an OD_600_ of 2.940 (Figure 2A).

#### 3.3.2 Carbon and nitrogen ratio effects

The effect of carbon and nitrogen ratios was assessed with media containing different glucose concentrations corresponding to the following C/N ratios: 0.5% (C/N = 0.625), 1.5% (C/N = 1.125), 3% (C/N = 1.875), and 4.5% (C/N = 2.625). The results showed that the highest bacterial growth occurred at 4.5% C/N ratio, with an OD_600_ value of 1.13 (Figure 2B).

#### 3.3.3 Temperature effect

**Figure 3:**
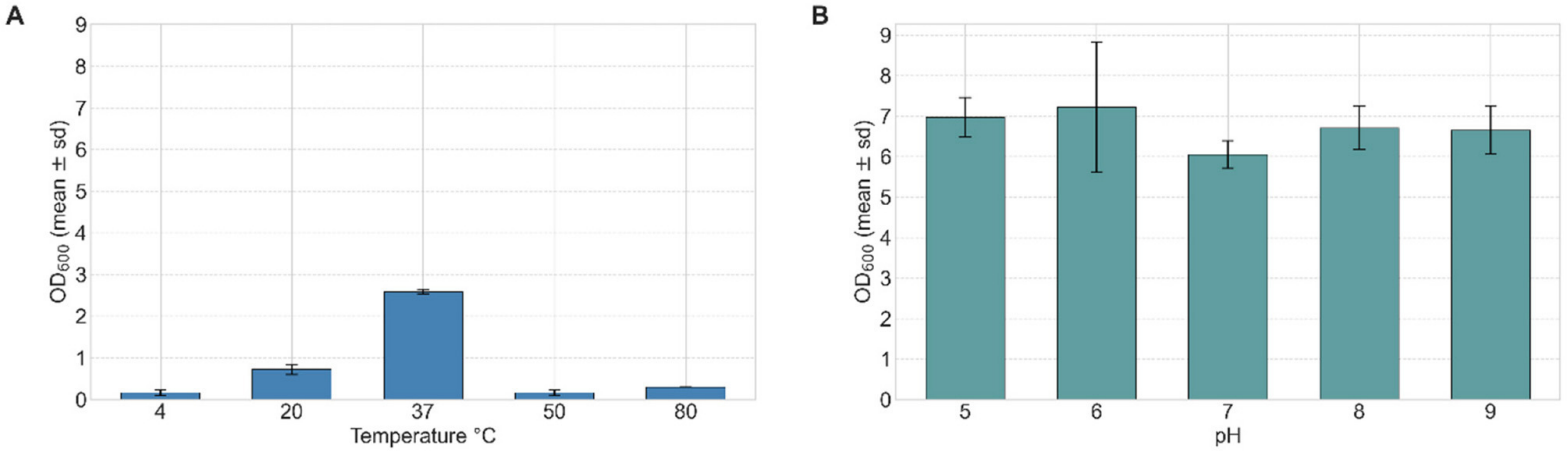
Effect of pH and temperature on the growth of strain VC-6. **(A)** The temperature effect in strain VC-6 increased OD_600_ up to 2.583 at 37°C after 24 h. **(B)** The highest OD_600_ reached 8.00 (with dilutions) at pH 6 after 24 h. Kruskal–Wallis test indicated a significant effect of pH on growth (OD_600_) [$\chi_{\left(4\right)}^{2}$ = 11.23, *p* = 0.024].

The effect of incubation temperature on strain VC-6 growth was evaluated at 4°C, 20°C, 37°C, 50°C, and 80°C. The optimal temperature was determined to be 37°C, yielding an OD_600_ of 2.583 (Figure 3A). Reduced growth was observed at the extreme temperatures of 4°C and 80°C, while moderate growth occurred at 20 and 50°C after 24 h of incubation.

#### 3.3.4 pH effect

The effect of initial pH on bacterial growth was investigated by adjusting the culture medium to pH values of 5, 6, 7, 8, and 9. Growth was measured through OD_600_ and cell dry weight after 24 h of incubation. Maximum growth was observed at pH 6, with an OD_600_ of 7.21 (Figure 3B) and a cell dry weight of 0.019 g mL^−1^, indicating optimal metabolic activity under mild acidic conditions.

#### 3.3.5 Fatty acids and triglycerides

**Figure 4:**
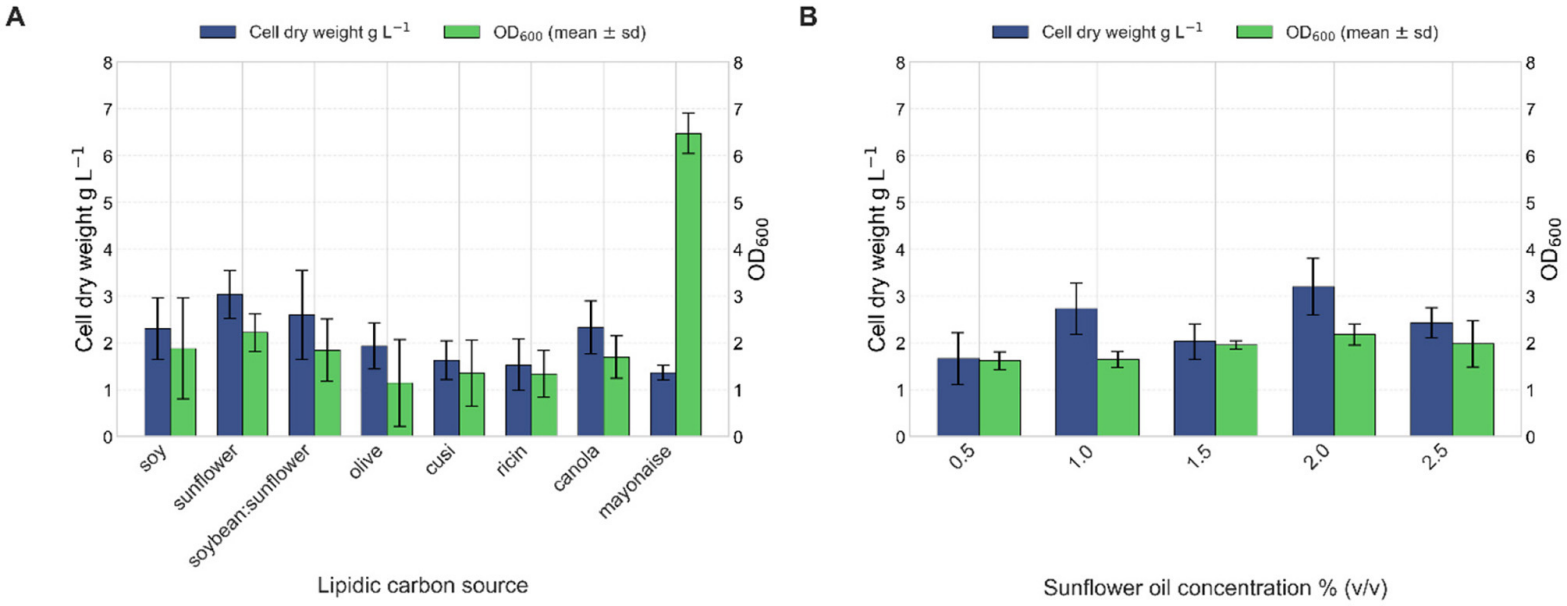
Effect of lipid source and sunflower oil concentration on the growth of strain VC-6. **(A)** Sunflower oil provided the highest growth reaching an OD_600_ of 2.22 after 24 h. Kruskal–Wallis test indicated a significant effect of sunflower as a lipidic source for cellular growth (OD_600_) [$\chi_{\left(15\right)}^{2}$ = 36.14, *p* = 0.0017]. **(B)** Maximum biomass production was observed at 2% (v/v) sunflower oil, reaching an OD_600_ of 2.18 and a cell dry weight of 3.2 g L^−1^.

The impact of various triglyceride and fatty acid sources on the growth of strain VC-6 was evaluated. The tested substrates included soybean oil, sunflower oil, a 1:1 mixture of soybean and sunflower oil, olive oil, cusi oil, castor oil, waste canola oil, and mayonnaise. Bacterial growth was quantified by OD_600_ and cell dry weight after 24 h. Sunflower oil provided the highest growth, with an OD_600_ of 2.22 and a cell dry weight of 3.0 g L^−1^ (Figure 4A). Additionally, this condition showed the lowest standard deviation among replicates, indicating consistent performance.

#### 3.3.6 Triglyceride concentration

Strain VC-6 was cultured in media containing 0.5%, 1%, 1.5%, 2%, and 2.5% (v/v) of the sunflower oil to determine the optimal growth concentration. Growth was assessed by OD_600_ and cell dry weight after 24 h. The highest biomass production occurred at 2% (v/v) sunflower oil, with an OD_600_ of 2.18 (Figure 4) and a cell dry weight of 3.2 g L^−1^, suggesting efficient metabolism of sunflower oil as a carbon and energy source.

### 3.4 Optimization of lipase production medium

#### 3.4.1 Determination of extracellular and intracellular lipase localization

The localization of lipase activity was assessed to determine whether the enzyme was present in the supernatant (extracellular activity), in the pellet (intracellular activity), or distributed between both intra- and extra-cellular compartments. Enzymatic activity assays were conducted using a combination of carbon sources in the form of triglycerides as the main substrate. Samples were separated into supernatant and pellet fractions by centrifugation, and enzymatic activity was measured fraction-wise.

Initial enzymatic assays show that extracellular activity is the highest enzymatic activity, with a general average of 2.019 U mL^−1^. Whereas, whole sample measurements provide only 1.968 U mL^−1^ of enzymatic activity, representing the combined extracellular and intracellular activity. Enzymatic assay of the bacterial pellet provided the lowest enzymatic activity, detected in this fraction, with a value of 1.676 U mL^−1^.

#### 3.4.2 Triglyceride-based carbon sources for lipase production

**Figure 5:**
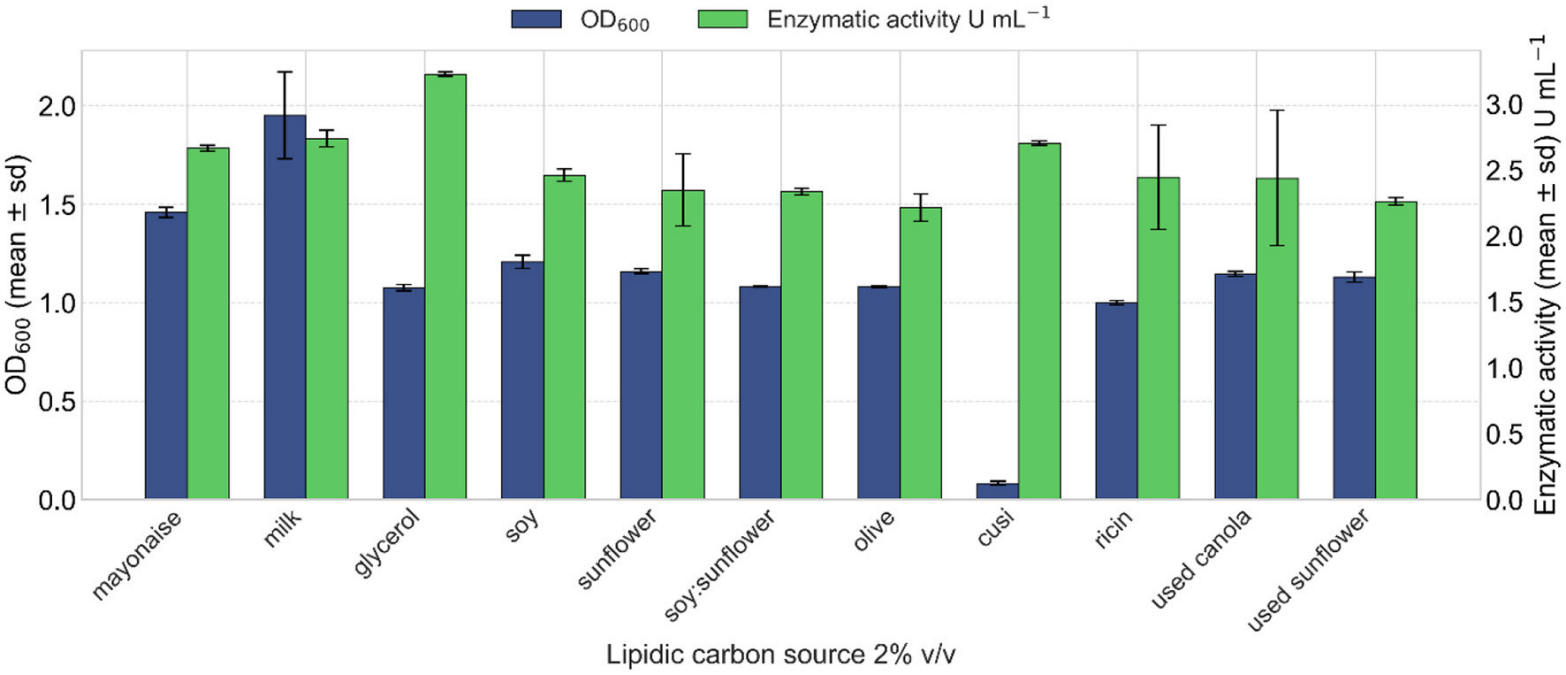
Effect of different triglyceride carbon sources on lipase production. Glycerol induced a lipase activity of 3.12 U mL^−1^. Kruskal–Wallis test indicated a non-significant overall effect of the TG source on lipase activity [$\chi_{\left(15\right)}^{2}$ = 21.19, *p* = 0.131].

The effect of different triglyceride-based carbon sources on lipase production of strain VC-6 was evaluated. Although fresh milk resulted in the highest OD_600_ (Figure 5), this outcome is not considered reliable due to the inherent turbidity of milk, which interferes with optical measurements and artificially elevates absorbance values (Sharma et al., 2001). Cell dry weight analysis revealed that olive oil supported the highest biomass production, reaching 5.8 g L^−1^. In terms of lipase production, the highest enzymatic activity was achieved when glycerol was used as the carbon source, yielding 3.12 U mL^−1^ of enzymatic extract.

**Figure 6:**
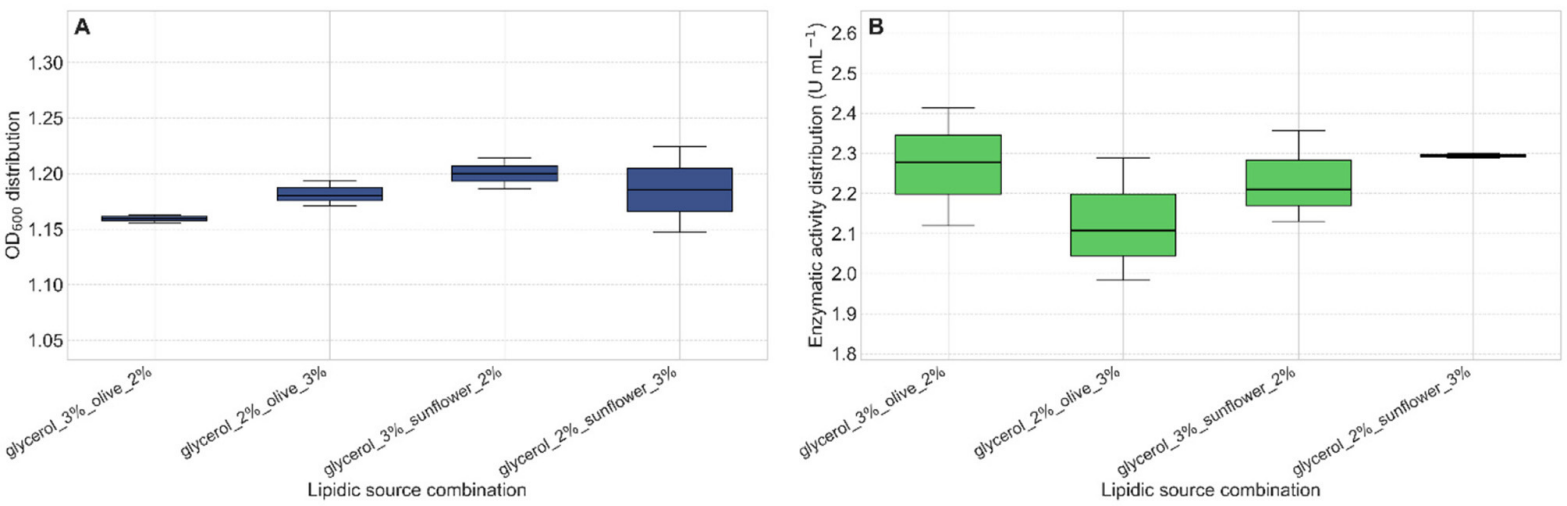
Effect of triglyceride-glycerol combinations on lipase activity. **(A)** Bacterial growth (OD_600_) after 24 h of incubation in media supplemented with different combinations of triglycerides and glycerol. **(B)** The combination of 3% (v/v) sunflower oil and 2% (v/v) glycerol resulted in the highest lipase activity (2.3 U mL^−1^) after 24 h.

Thereafter, a combination of the carbon sources sunflower oil and glycerol resulted in the highest levels of both biomass production and lipase activity. Specifically, the mixture containing 3% (v/v) sunflower oil and 2% (v/v) glycerol produced the maximum lipase activity, reaching 2.3 U mL^−1^ after 24 h of incubation (Figure 6).

#### 3.4.3 Nitrogen sources effect on enzyme production

**Figure 7:**
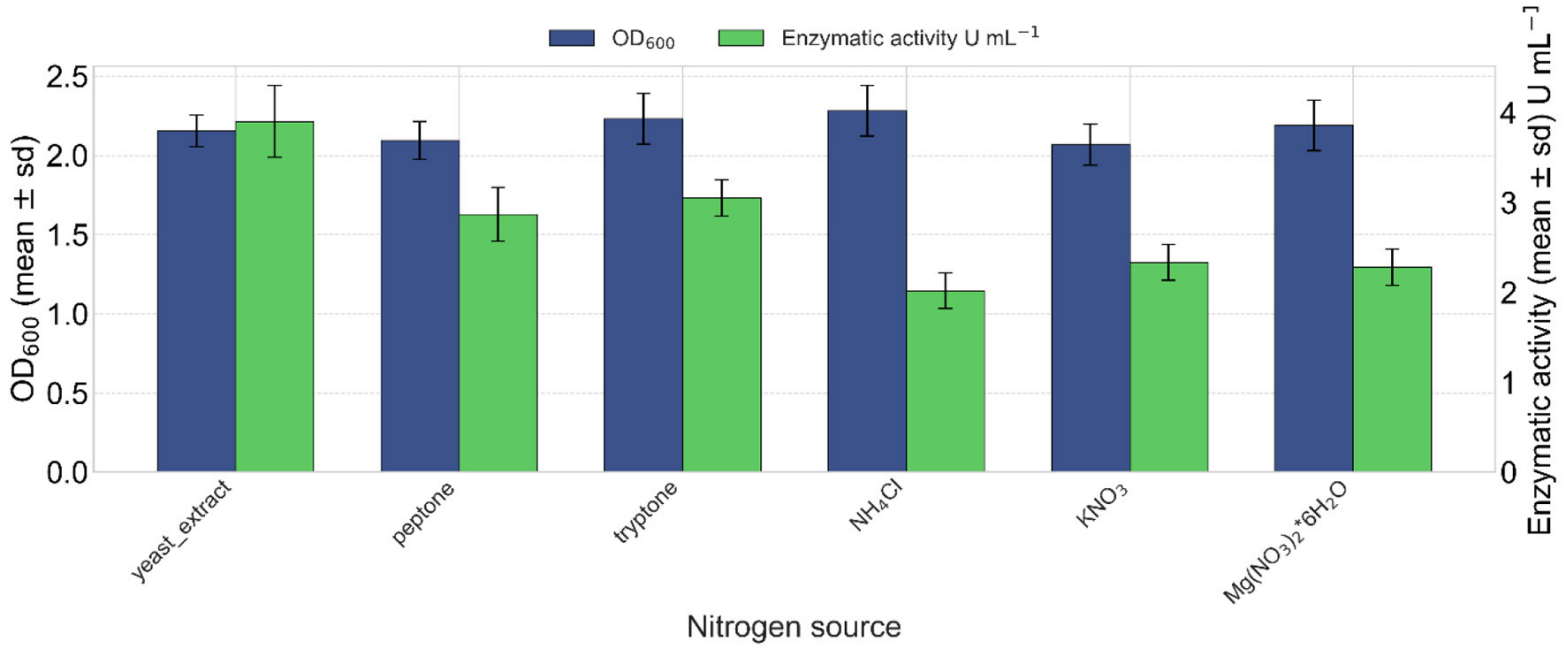
Effect of different nitrogen sources on lipase production. Ammonium chloride resulted in the highest cellular growth (OD_600_ values in blue). Kruskal–Wallis test indicated a non-significant effect of the nitrogen source on growth (OD_600_) [$\chi_{\left(5\right)}^{2}$ = 4.72, *p* = 0.451]. In green, yeast extract induced the highest lipase activity (3.90 U mL^−1^). Kruskal–Wallis test indicated a significant effect of the nitrogen source on lipase activity [$\chi_{\left(5\right)}^{2}$ = 15.18, *p* = 0.0096].

Yeast extract (1% w/v) was the optimal nitrogen source for lipase production after 24 h of incubation, reaching a maximum enzymatic activity of 3.90 U mL^−1^ of enzymatic extract at 24 h (Figure 7). However, in terms of OD_600_ density, ammonium chloride yielded a slightly higher value, followed by magnesium nitrate hexahydrate (Figure 7).

#### 3.4.4 Inorganic salts

**Figure 8:**
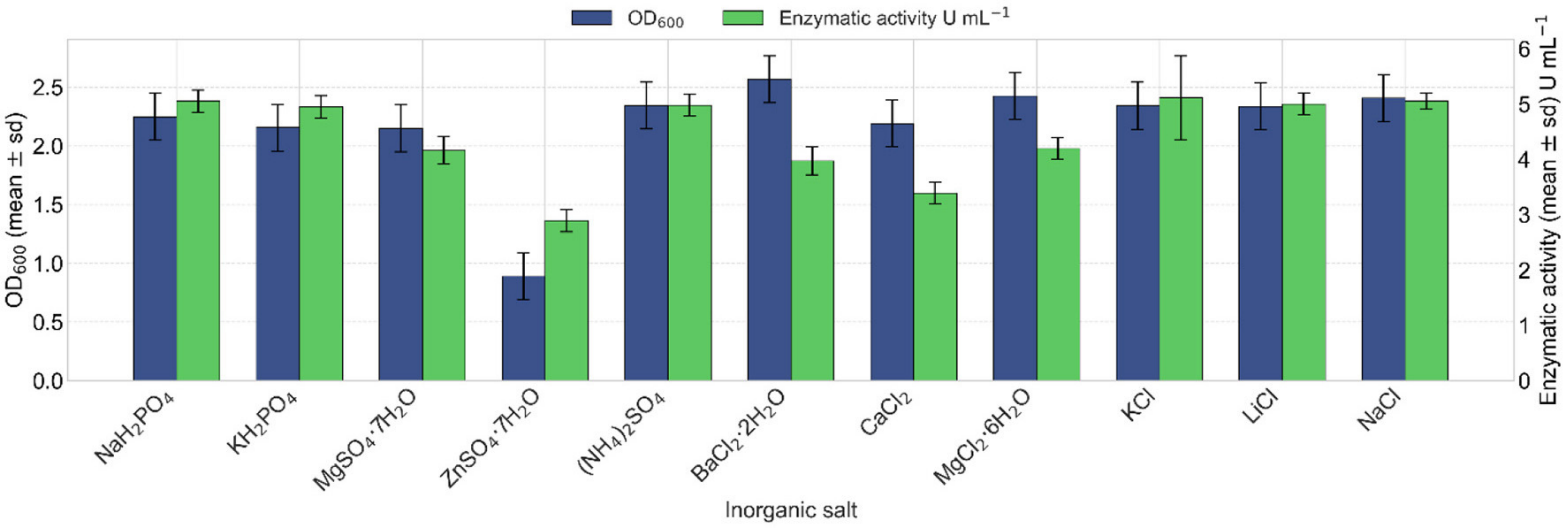
Effect of different inorganic salts on lipase production. Barium chloride dihydrate provided the highest cellular growth (OD_600_ values in blue). Kruskal–Wallis test indicated a non-significant overall effect of the salt type on growth (OD_600_) [$\chi_{\left(10\right)}^{2}$ = 16.12, *p* = 0.096]. Highest lipase activities (in green) were induced by potassium chloride and sodium chloride, reaching 5.44 and 5.15 U mL^−1^, respectively. Kruskal–Wallis test indicated a significant effect of the salt type on lipase activity [$\chi_{\left(10\right)}^{2}$ = 26.35, *p* = 0.0033].

The effect of different inorganic salt sources on lipase production of strain VC-6 was assessed after 24 h of incubation. Among the tested salts, sodium chloride (NaCl) and potassium chloride (KCl) exhibited the highest enzymatic activities, with values of 5.15 and 5.44 U mL^−1^, respectively (Figure 8). Regarding bacterial growth, cell dry weight measurements indicated that the highest biomass was obtained with calcium chloride (CaCl_2_) and sodium chloride (NaCl). Meanwhile, OD_600_ revealed enhanced growth in the presence of barium chloride dihydrate (BaCl_2_·2H_2_O), followed by sodium chloride (NaCl) (Figure 8).

#### 3.4.5 Concentration of selected inorganic salt

**Figure 9:**
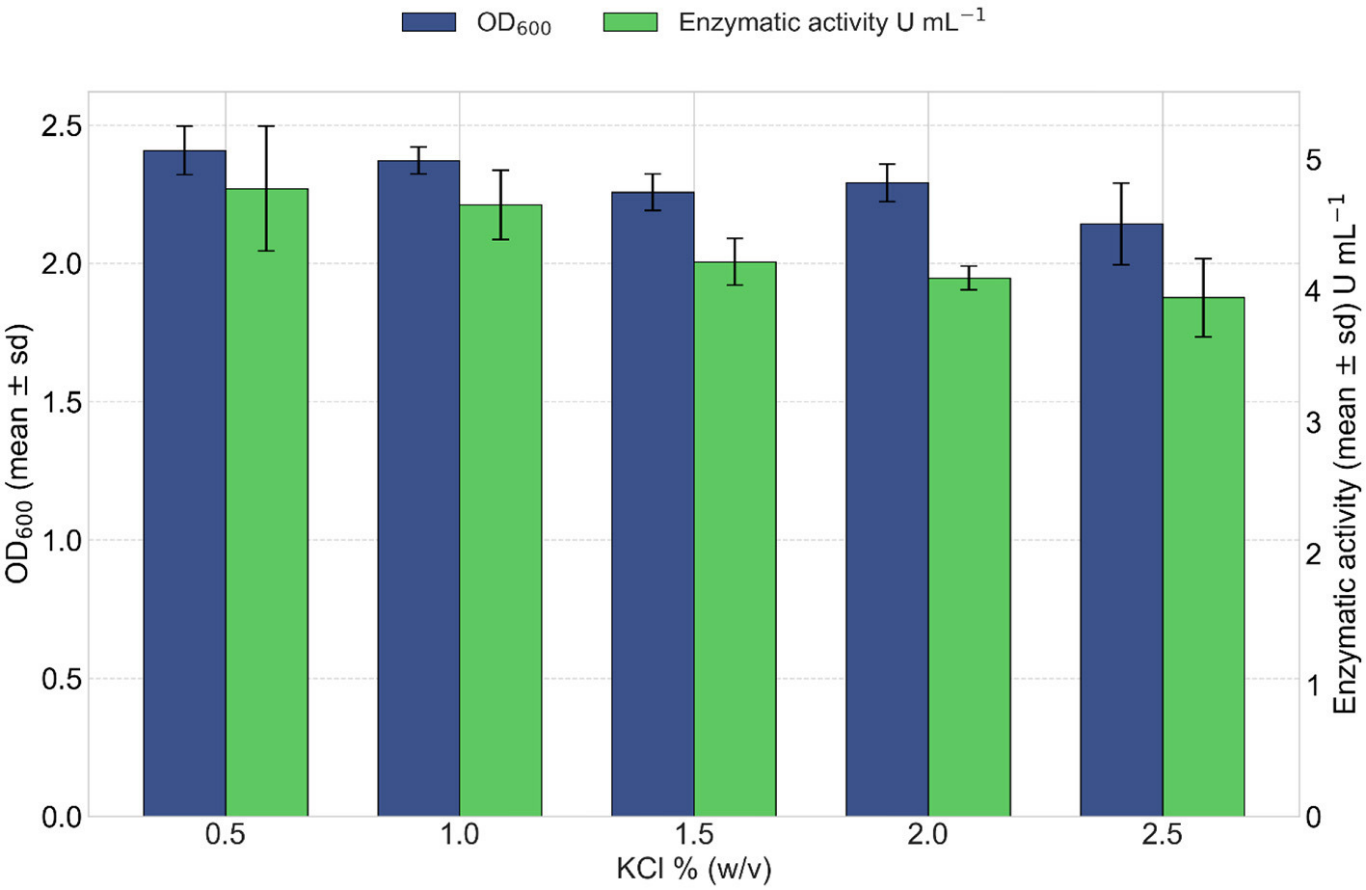
Effect of KCl concentration on lipase production. After 24 h of incubation with increasing concentrations of KCl, a gradual decrease in cellular growth (OD_600_ values in blue) was observed with higher salt concentrations. Kruskal–Wallis test indicated a significant effect of salt concentration on growth (OD_600_) [$\chi_{\left(4\right)}^{2}$ = 9.53, *p* = 0.049]. Lipase activity (in green) peaked at 0.5% KCl and declined progressively at higher concentrations. Kruskal–Wallis test indicated a marginally significant effect of KCl on lipase activity [$\chi_{\left(4\right)}^{2}$ = 9.48, *p* = 0.050].

The effect of different concentrations of potassium chloride (KCl) previously identified as the optimal monovalent cation source was evaluated for its effect on lipase production and bacterial growth over a 24 h period. The highest lipase production (4.77 U mL^−1^) was achieved at a concentration of 0.5% (w/v) KCl (Figure 9). At this concentration, maximum bacterial growth was also recorded (OD_600_ reaching 2.41 at 24 h), as shown in Figure 9. Higher KCl concentrations (1%, 1.5%, 2%, and 2.5% w/v) yielded a gradual decline in both enzymatic activity and bacterial growth.

#### 3.4.6 Culture medium pH

**Figure 10:**
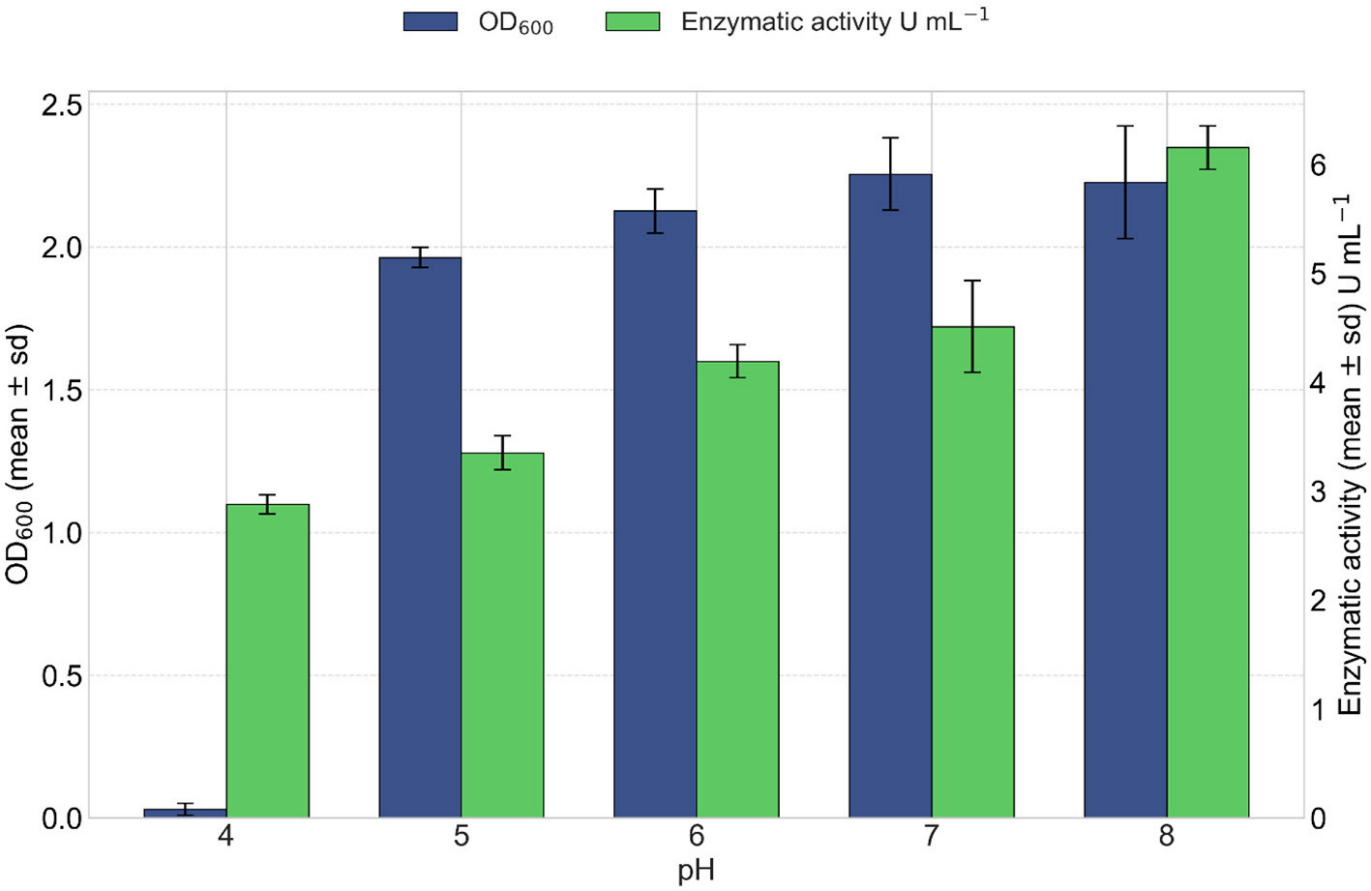
Effect of pH on lipase production. Bacterial growth (in blue) increased steadily with pH. Optimal values observed between pH 6 and 8. Kruskal–Wallis test indicated a significant effect of pH on growth (OD_600_) [$\chi_{\left(4\right)}^{2}$ = 11.23, *p* = 0.024]. Lipase activity (in green) increased under alkaline conditions, reaching a maximum at pH 8. Kruskal–Wallis test indicated a significant effect of pH on lipase activity [$\chi_{\left(4\right)}^{2}$ = 12.98, *p* = 0.011].

pH effect on enzymatic activity and growth of the VC-6 strain was evaluated after 24 h of incubation. The results showed that pH has a significant impact on both bacterial growth and lipase production. Bacterial growth, assessed by both cell dry weight and OD_600_, was consistently high at pH values of 6, 7, and 8, indicating that these conditions are optimal for VC-6 strain proliferation, as observed in Figure 10. However, at pH 4 and 5, growth decreased markedly. Although growth remained satisfactory across pH 6–8 range, maximum lipase production was observed only at pH 8, suggesting that enzyme biosynthesis is more sensitive to pH than cell growth. The highest enzymatic activity was recorded at pH 8, with a value of 6.15 U mL^−1^ of enzymatic extract as observed in Figure 10.

### 3.5 Genetic identification and characterization

#### 3.5.1 Molecular identification

Taxonomic identification of strain VC-6, based on full-length 16S rRNA gene sequencing, demonstrated 99.93% identity to *Bacillus safensis*, supporting its classification within this species.

**Figure 11:**
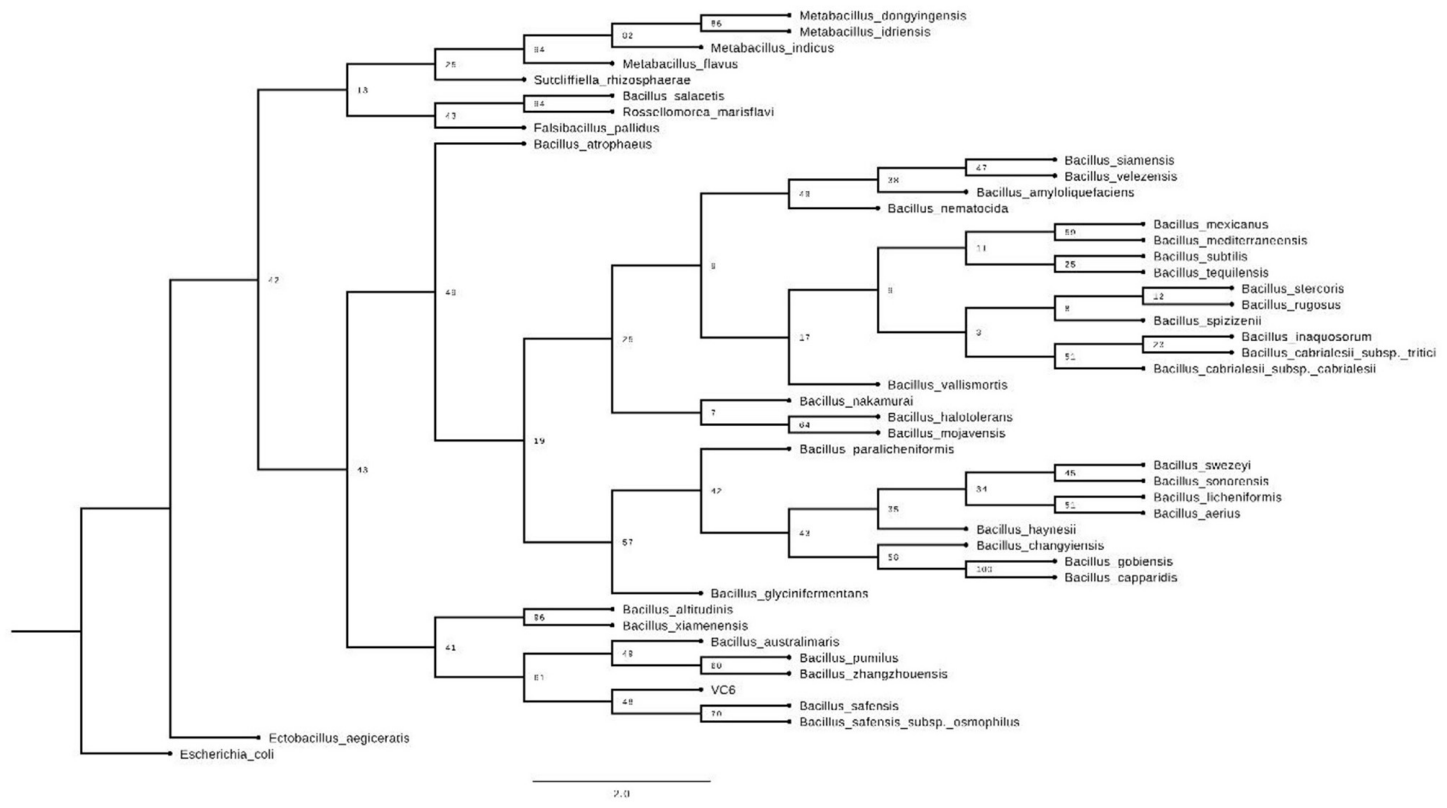
Phylogenetic tree of strain VC-6 in relation to *Bacillus safensis* and other related microorganisms.

When inferring the phylogenetic tree, it was observed that this strain clusters closely with other members of the genus *Bacillus*, particularly *Bacillus safensis* (Figure 11). However, it positions itself in a separate clade, which could suggest subtle but significant differences in its sequence or recent evolution (Arevalo et al., 2022; Salwan and Sharma, 2022).

#### 3.5.2 Assembly and functional annotation

The assembly resulted in a genome of 2,199,211 base pairs, with an N50 of 100,000 bp and 56 contigs. This process enabled the identification and characterization of 3,449 predicted proteins, among which three lipases of particular interest were identified: Phospholipase YtpA, a protein ~30 kDa in size; Spore Germination Lipase LipC, a protein ~25 kDa in size; and Thermostable Monoacylglycerol Lipase, a protein ~28 kDa in size.

### 3.6 Lipase production under controlled bioreactor conditions

**Figure 12:**
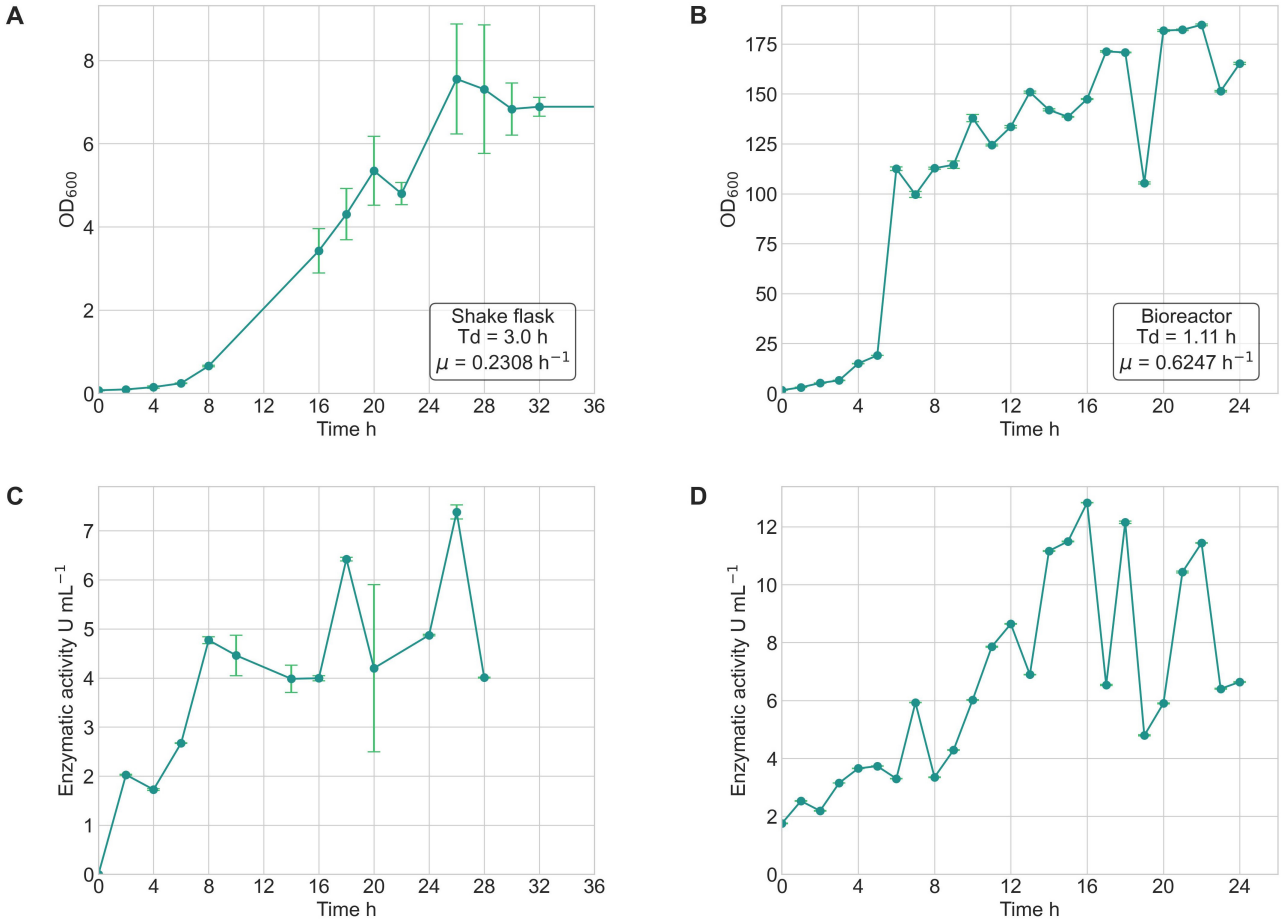
Growth kinetics and lipase activity of *Bacillus safensis* VC-6 in shake flasks and bioreactor cultures. **(A)** Bacterial growth curve (OD_600_) in shake flasks, showing a specific growth rate (μ) of 0.2308 h^−1^ and a doubling time (T_d_) of 3.0 h. **(B)** Growth curve in the bioreactor, with a higher μ of 0.6247 h^−1^ and Td of 1.11 h. **(C)** Lipase activity in shake flasks revealed three peaks at 8, 18, and 26 h, with a maximum activity of 7.38 U mL^−1^. **(D)** In the bioreactor, three peaks were observed at 16, 18, and 22 h, with a maximum of 12.83 U mL^−1^.

The comparative analysis between shake flask and bioreactor cultures of *Bacillus safensis* VC-6 revealed notable differences in both bacterial growth kinetics and lipase production profiles. In shake flasks, the strain exhibited a specific growth rate (μ) of 0.2308 h^−1^, with a doubling time (T_d_) of 3 h, reaching the stationary phase after ~24 h and a maximum OD_600_ of 8 (Figure 12A). Additionally, cultures grown in the bioreactor showed higher specific growth rate (μ) 0.6247 h^−1^ and doubling time (T_d_) 1.11 h, achieving an OD_600_ above 140 in just 15 h (Figure 12B), indicating enhanced biomass accumulation under controlled conditions.

Lipase production in shake flasks presented three distinct activity peaks at 8 h (4.71 U mL^−1^), 18 h (6.42 U mL^−1^), and 26 h (7.38 U mL^−1^), corresponding to the phases of bacterial growth (Figure 12C). Similarly, the bioreactor culture also displayed three well-defined peaks of enzymatic activity at 16 h (12.83 U mL^−1^), 18 h (12.15 U mL^−1^), and 22 h (11.44 U mL^−1^) (Figure 12D). In both systems, enzyme activity increased in parallel with biomass accumulation, suggesting that lipase production is growth-associated.

### 3.7 Lipase activity control in optimized production medium

The internal controls validated the experimental system. Strain VC-6 demonstrated a higher total protein concentration than commercial lipase control, confirming its capacity to secrete substantial quantities of enzymatic proteins under optimized conditions. Furthermore, enzymatic activity derived from VC-6 consistently exceeded that of the commercial control throughout the assay, while the negative control exhibited no significant activity, as expected.

## 4 Discussion

The present study focused on the optimization of lipase production from the strain *Bacillus safensis* VC-6, isolated from the unique and extreme environment of the Copahue volcanic region, Chile. This strain demonstrated extremophilic characteristics in the initial screening stages. The strains capability to grow up to 10% (w/v) NaCl and surpassing growth temperatures of 50°C (data not shown) strongly suggest VC-6 extremophilic classification. Bioprospecting in extreme ecological niches, such as this region and other singular environments including salt flats, hot springs, deserts, hypersaline sediments, and high-altitude brackish water lakes has been consolidated as an exceptionally fruitful strategy for the discovery of enzymes with novel and robust characteristics (López et al., 2023; Moreno et al., 2013; Ruginescu et al., 2020; Lestari et al., 2015; Khodaverdi and Ghassemian, 2018; Essamri et al., 2018; Mohamed and Awad, 2021; Khunt et al., 2013; Al-Daghistani et al., 2024; McGonigle et al., 2022; Devi et al., 2025). These enzymes are of significant interest due to their potential industrial and pharmaceutical applications, as microorganisms adapted to extreme environments are able to produce intrinsically robust enzymes, with tolerance to adverse conditions that confers biotechnological industrial value (Ashaolu et al., 2024; Qiu et al., 2021; Verma et al., 2021; Myers et al., 2013; Moreno et al., 2013; Yavari-Bafghi and Amoozegar, 2025). Food industry and pharmaceutics require diverse solutions provided by findings in bacterial extremophilic enzymes (Yao and Shuangping, 2023; Yavari-Bafghi and Amoozegar, 2025). The search for extracellular enzymes is particularly relevant for industrial-scale processes, such as the lipase produced by strain VC-6, given that their secretion into the culture medium facilitates subsequent recovery and purification steps (Drissi Kaitouni et al., 2020).

Sequential optimization of culture conditions revealed a clear dissociation between the optimal conditions for cellular growth and those maximizing extracellular lipase production. Optimal cellular growth was found to be achieved at pH 6 and 37°C, and sunflower oil provided the highest growth. Furthermore, ammonium chloride and barium chloride dihydrate provided the highest mean OD_600_ values. However, increasing concentrations of KCl resulted in a significant decrease in cellular growth.

In contrast to growth, maximal extracellular lipase production was obtained at an alkaline pH of 8, while maintaining the temperature at 37°C. This difference observed between growth and production optimal pH suggests the existence of specific metabolic regulation directing enzyme biosynthesis and production (Drissi Kaitouni et al., 2020). The alkaline optimal pH for VC-6 lipase production is a common characteristic among many bacterial lipases and, notably, aligns with the properties of halophilic enzymes, which often exhibit not only activity under alkaline conditions but also salt-dependent stability and tolerance to organic solvents, highly desirable properties for diverse industrial and pharmaceutical applications (Amoozegar et al., 2008; Lestari et al., 2015; Khodaverdi and Ghassemian, 2018; Essamri et al., 2018; Mohamed and Awad, 2021; Qiu et al., 2021; Yavari-Bafghi and Amoozegar, 2025). Other studies on *B. safensis* have also reported optimal activity for its enzymes from neutral to alkaline conditions, such as pH 7.0 for a lipase (Si et al., 2023), pH 8.0 for a serine peptidase (Wang et al., 2025), and pH 9 for a triacylglycerol lipase (Wang et al., 2024). However, the optimal production temperature (37°C) classified the enzyme produced by VC-6 as mesophilic, which contrasts with the intrinsically thermostable or thermotolerant lipases produced by some other extremophiles that operate and are stable at higher temperatures (Khodaverdi and Ghassemian, 2018; Essamri et al., 2018; Amoozegar et al., 2008; Qiu et al., 2021). The specific thermostability of the VC-6 lipase(s) under active reaction conditions still requires detailed characterization of enzymatic activity stability and biochemical elucidation, which will be performed in future studies.

The specific composition of the culture medium determines lipase production induction and optimization (Sharma et al., 2001). The addition of 1% (w/v) yeast extract was identified as an effective nitrogen source for lipase production. While the synergistic combination of sunflower oil at 3% (v/v) and glycerol at 2% (v/v) was determined to be the optimal inducer/carbon source combination, achieving the highest lipase production (de Almeida et al., 2013). However, the overall effect of the TG source type on lipase activity was found to be non-significant. This suggests that while certain TG sources or combinations may lead to higher absolute activity levels, the variation among the tested TG sources as a group did not reach statistical significance. The induction of bacterial lipase production through the addition of vegetable oils is a well-established strategy (Amoozegar et al., 2008; Mohamed and Awad, 2021). In this study, glycerol, which in the carbon source optimization tests for production resulted to be the best individual inducer among the substrates tested, could potentially enhance both the specific induction of lipolytic genes and provide a readily available carbon source for energy metabolism of the strain, thereby supporting increased enzyme synthesis.

A particularly interesting result was the effect of salt addition to the medium. While the overall effect of different salt types on lipase activity was significant, with KCl and NaCl inducing the highest activities (5.44 and 5.15 U mL^−1^, respectively). Conversely, lipase activity peaked at 0.5% (w/v) KCl and declined progressively at higher concentrations. This behavior is a distinctive characteristic of strain VC-6 and its lipase production, as it differs markedly from typical halophilic lipases that not only require, but are often dependent on high salt concentrations to maintain their structure, activity, and stability (Essamri et al., 2018; Khunt et al., 2013; Amoozegar et al., 2008; Qiu et al., 2021; Yavari-Bafghi and Amoozegar, 2025). This phenomenon of enzyme production modulation by ionic strength (Drissi Kaitouni et al., 2020) underscores the complexity of microbial adaptation mechanisms to extreme environments, especially those with fluctuating salinity like the Copahue volcanic region. It could be suggested that the adaptation of *Bacillus safensis* VC-6 to its environment of origin may involve specialized osmotic stress management mechanisms, such as the production or accumulation of compatible solutes or specific ion transport systems, which might indirectly affect enzyme expression and production in response to external salinity (Al-Daghistani et al., 2024).

Lipase production in a bench-top bioreactor, under the flask-optimized conditions, reached a significant level, obtaining a maximal activity of 12.83 U mL^−1^ at 16 h of cultivation. This yield is comparable to or higher than that reported for various other *Bacillus* species and other lipase-producing microorganisms under optimized culture conditions. For example, a production of 1.82 U mL^−1^ is reported for *Halobacillus truperi* AR11 (Khodaverdi and Ghassemian, 2018) under optimized conditions. Furthermore, the enzymatic activity values obtained for strain VC-6 (12.83 U mL^−1^) are within the range reported for other *Bacillus* sp. lipase producers, such as those studied by Szymczak et al. (2021), who reported maximal activities of 12–18 U mL^−1^ under optimized conditions with similar strains. This scale-up to bioreactor demonstrated the process viability and the critical importance of precise control over key environmental parameters like pH and dissolved oxygen to achieve high production levels in a controlled system (Drissi Kaitouni et al., 2020). Future investigations could focus on employing more sophisticated statistical experimental designs, e.g., Surface Response Methodology, to further refine culture conditions and increase VC-6 lipase yields (Karkouri et al., 2019; Mohamed and Awad, 2021; Bandal et al., 2021).

Genetic identification of the strain, performed by 16S rRNA gene sequence analysis, confirmed its belonging to the species *Bacillus safensis* (Lateef et al., 2015), a species recognized for its remarkable metabolic robustness and its ability to colonize diverse ecological niches, including environments associated with space activities due to its resistance to dehydration and radiation. *In silico* functional genomic analysis of strain VC-6 revealed the presence of key genes presumably involved in lipid metabolism and lipase production. Genes encoding a phospholipase (*YtpA*) and a germination lipase (*LipC*) were identified, enzymes that generally participate in the hydrolysis of phospholipids and triacylglycerols during processes like spore germination or the utilization of lipids as an energy source. Notably, and particularly promising for industrial applications, the genomic analysis also identified a putative gene for a thermostable monoacylglycerol lipase (MAG), which is a strong indication of this strain's potential to produce an enzyme with high thermal stability. Thermostable MAG-type lipases are highly valued enzymes in industry due to their substrate specificity toward monoacylglycerols and their inherent stability at high temperatures, making them ideal catalysts for lipid synthesis and modification processes (Ashaolu et al., 2024; Verma et al., 2021; Myers et al., 2013), such as the production of functional mono- and diacylglycerols or the synthesis of lipid-relevant compounds for drug delivery (Yavari-Bafghi and Amoozegar, 2025).

Although detailed biochemical characterization of the enzyme encoded by this gene was not addressed in the present study, the presence of a gene encoding a putative thermostable monoacylglycerol lipase (MAG) supports the hypothesis that this enzyme might possess structural adaptations typically found in extremozymes. These include increased proportion of proline residues (restrict conformational flexibility), enhanced salt bridge networks, compact hydrophobic cores, and reduced surface-exposed polar residues, all of which contribute to increased thermal stability and resistance to denaturation (Santiago et al., 2016; Siddiqui and Cavicchioli, 2006). In addition, thermostable enzymes often exhibit a greater number of ionic interactions and improved secondary structure packing, such as higher α-helix content (Vieille and Zeikus, 2001). Although a detailed structural analysis was beyond the scope of the study, future work will include *in silico* 3D structure prediction, molecular dynamics simulation and laboratory tests to validate the thermostability potential of this lipase (Littlechild, 2015).

The potential tolerance to organic solvents, suggested both by the strain's origin from an extreme environment and by the expected robustness of *B. safensis* enzymes, significantly broadens its application potential in reactions conducted in non-aqueous media or in the presence of organic solvents, such as biodiesel synthesis, the production of active pharmaceutical ingredients, or lipid-based pharmaceutical formulations (Essamri et al., 2018; Amoozegar et al., 2008; Moreno et al., 2013; Lestari et al., 2015; Karkouri et al., 2019; Yavari-Bafghi and Amoozegar, 2025). Thus, the genomic data obtained provided a crucial molecular basis that complemented and reinforced the physiological production observations and the putative enzyme properties, offering a solid foundation for future investigations and biotechnological applications. Future studies involving differential expression or targeted mutagenesis are suggested to confirm the hypothesized direct relationship between the identified genes and lipase production.

## 5 Conclusion

*Bacillus safensis* VC-6, an extremophilic strain from Copahue volcanic soils, was identified and its potential as a promising source of robust lipases was established. Culture conditions were optimized to maximize bacterial growth and lipase production. The process was carried out in a bioreactor, where multiple activity peaks were observed and the highest enzymatic activity reached 12.83 U mL^−1^. Genetic identification confirmed the strain's identity and its extremophilic origin. Functional genomic analysis revealed the presence of multiple key lipolytic genes, including *YtpA, LipC*, and a putative thermostable MAG-type lipase. The identification of such critical lipase genes provides a molecular explanation for the enzymes' stability and activity profiles. These findings establish *B. safensis* VC-6 as a highly valuable candidate for industrial applications that require stable enzymes. Future projections include the purification and detailed characterization of the individual lipases identified genomically. Cloning and heterologous expression of the identified genes is also contemplated to further optimize production. Validating its performance in specific industrial processes will be essential. This strain opens a significant path toward the development of novel, robust, and efficient biocatalysts under industrially relevant conditions.

## Data Availability

The original contributions presented in the study are publicly available. This data can be found here: https://www.ncbi.nlm.nih.gov/genbank/, accession number PX056115.
